# Perception of Ghanaian Medical Students of Cadaveric Dissection in a Problem-Based Learning Curriculum

**DOI:** 10.1155/2018/3868204

**Published:** 2018-07-05

**Authors:** Abass Alhassan, Saeed Majeed

**Affiliations:** ^1^Anatomy Department, School of Medicine and Health Sciences, University for Development Studies, Tamale, Ghana; ^2^Physiology Department, School of Medicine and Health Sciences, University for Development Studies, Tamale, Ghana

## Abstract

There is a drastic shift in medical curriculum from the traditional medical curriculum where various basic science disciplines are taught separately in the preclinical years to integrated problem-based learning (PBL) in many medical schools across the world. In the integrated PBL, the time for classical anatomy dissection is significantly reduced. There are varying views across the world about the perception of medical students to cadaveric dissection. There is however no research on student's perception of cadaveric dissection in Ghana. The present study was to assess Ghanaian medical student's perception of cadaveric dissection under the PBL curriculum and to assess which educational tool students rely on to study anatomy. An anonymous self-administered, Likert-style questionnaire consisting of 24 questions was administered to 132 second- and third-year students after they had completed the dissection schedules for the musculoskeletal system. Participation was voluntary. In all, 89.5% of the students indicated that they had attended all the dissection sessions. The students generally agreed that dissection deepens their understanding of anatomy (87.9%), provides better understanding of clinical skill examination (66.7%), enhances their respect towards the human body (66.6%), provides better understanding of the effect of trauma (69.7%), and makes learning interesting (90.9). However, 57.5% of them agreed or strongly agreed that dissection was stressful. Majority of the students also disagreed that dissection should be eliminated from the curriculum (100%). This study has shown a strong positive perception towards the use of cadaveric dissections in teaching and learning anatomy regardless of the fact that SMHS/UDS uses the integrated PBL curriculum.

## 1. Introduction 

Medical education all over the world has seen a drastic shift from the traditional methods of teaching and learning where various basic science disciplines are taught separately in the preclinical years, to integrated teaching and learning with clinical cases at the center of learning. Traditionally, anatomy has been taught using different approaches including didactic lectures, practical sessions based on models, prosected materials, and cadaveric dissection as well as newer methods such as computer-assisted learning models and interactive computer-based software and radiological images. Cadaveric dissection has been one of the key modes of delivering an anatomy curriculum in medical schools across the world for many years. However, there has been a shift in medical education pedagogy from the more lectures centered around problem-based learning (PBL) or case-based approaches [1]. These new curricula according to the proponents are aimed at using real-life patients' cases as the stimuli for learning in order to integrate basic and clinical science [2].

An integrated medical curriculum refers to a noncompartmentalized approach to basic sciences whereby lectures on subjects like embryology, histology, anatomy, physiology, and pathology are spread out over the course of the first two or three years [3]. It is usually organized around a clinical scenario involving organ systems. ACMI-TRI project report [4] and other recommendations [5–7] propose the need for greater integration of subjects in the medical curriculum [8].

In Ghana, undergraduate medical training consists of three years of basic science training and three years of clinical training. The teaching and learning methodology is largely a conventional discipline, lecture-based approach. However, the School of Medicine and Health Sciences, University for Development Studies, uses the PBL pedagogy in the training of medical students [9].

The use of PBL pedagogy is to enhance lifelong learning with a focus on main principles rather than minute details and to expose students to hospital environment earlier as well as providing them with enhanced communication skills. It also encourages collaborative learning and self-directed learning. In addition, PBL encourages the use of information technology as a means of curriculum delivery and the use of a wide range of learning resources in the curriculum. These changes have resulted in a drastic reduction of time available for the teaching of anatomy through cadaveric dissections and sometimes complete elimination of this teaching approach [10–12].

A good anatomical knowledge is fundamental and indispensable to efficient and safe clinical practice and for the understanding of other subject disciplines such as physiology, pathology, and surgery. Moreover, studies have shown that anatomical dissections reinforce respect and compassion among medical students [13]. The high costs, time intensity, the requirement for highly skilled teachers, and the emotionally challenging nature of cadaveric dissection as well as being a cause of significant psychological distress among medical students have been cited as its potential disadvantages [14].

Although there have been a number of studies in literature looking at the perception of students of the use of anatomical dissection as a means of delivering anatomy knowledge, they cannot however be used to describe exactly the situation in Ghana owing to the heterogeneity of study methodologies and the variation in general expectations and perception of students in different geographical settings. The purpose of this study, therefore, was to explore student perception of anatomy dissection in a Ghanaian university with a PBL-based curriculum.

## 2. Methods

### 2.1. Setting

The study was carried out at the Department of Anatomy, School of Medicine and Health Science of the University for Development Studies. The school is the only medical school in Ghana using a fully integrated PBL curriculum which comprises three years of preclinical training and three years of clinical training. It emphasized small-group, student-centered, self-directed learning of basic and clinical sciences. The structure of the PBL curriculum in SMHS and UDS has been well documented by Mogre et al. [9]. Briefly, teaching and learning are organized around discipline integrated blocks of 4–6-week duration. The objectives of each block are met through tutorials, skills training, practical laboratory training, and lectures. The lectures are meant to clarify concepts from the tutorials that the students did not understand or that were part of the difficult subject matter. One of the major blocks of year two is the musculoskeletal system block. Previously only prosected materials were used in the teaching and learning of anatomy. However, the last two years had seen students performing dissection under the supervision of a tutor/lecturer 4 hours per week for the duration of the block. The students also have access to audio/visual material on dissection prior to the actual dissection period.

### 2.2. Subjects

The study population consisted of second- and third-year medical students of the School of Medicine and Health Sciences of the University for Development Studies. One hundred and thirty-two students (66 second-year and 66 third-year students) completed an anonymous voluntary questionnaire at the end of the musculoskeletal dissection module.

The questionnaire was a self-administered, Likert-style instrument consisting of 24 questions adapted from the study of Dissabandara et al. [15] and Azer et al. [16]. The questions addressed four broad areas with questions evaluating positive experiences, negative experiences, and questions comparing dissection with other forms of learning as well as questions about the preferred mode of learning anatomy. For each of the questions, students were to indicate whether they strongly disagree, disagree, neutral, agree, or strongly agree by choosing 1, 2, 3, 4, or 5, respectively.

The data were analyzed using SPSS Version 25.0 for the production of descriptive statistics in which the frequency of the replies was determined for each item of the questionnaire.

## 3. Results

The results of the student perception survey are summarised and presented in Tables 1, 2, 3, and 4. Of the 132 students who completed the questionnaire, 85 (64.4%) were males and 47 (35.6%) were females. In all, 89.5% of the students indicated that they had attended all the dissection sessions. Majority of the students (77.3%) agreed or strongly agreed that they were satisfied with the dissection program. The students agreed or strongly agreed that dissection deepens their understanding of anatomy (87.9%), provides better understanding of clinical skills examination (66.7%), enhances their respect towards the human body (66.6%), provides better understanding of the effect of trauma (69.7%), and makes learning interesting (90.9%). They also related to the statement that dissection helps them to recall what they have learned (87.0%) and gives them lasting knowledge (65.2%) as shown in Table 1.

Over half of the respondents agreed or strongly agreed that it was difficult to locate structures but disagreed with the proposition that they could not differentiate between structures. They also disagreed that they were bored with the way the dissection was carried out. However, 57.5% of them agreed or strongly agreed that dissection was stressful. Majority of the students also disagreed or strongly disagreed to statements such as “dissection should be eliminated from the curriculum” (100%), “dissection is against my religion” (95.4%), and “dissection is against my culture” (97.0%). Almost all the students were unanimous that they did not like the smell of the formalin (Table 2).

In comparing cadaveric dissection to other forms of learning, about 75.0% of the students disagreed or strongly disagreed that dissection should be replaced by lectures, computer-based learning, or prosecuted materials. The majority (70%) of them agreed or strongly agreed that they prefer dissection over other forms of learning and that more time should be allocated to dissection. Over 90% of them also agreed or strongly agreed that they would be disadvantaged if they do not attend dissection class.

In analyzing the preferred mode of learning anatomy, dissection emerges as the most preferred tool for learning anatomy with 53.0% of the second-year and 48.5% of the third-year students saying they prefer dissection over the others. The second most preferred mode of learning was lectures with 15.1% and 21.2% of the second-year and third-year students, respectively. This was followed by self-study with 10.6% for second-year students and 13.6% for third-year students. Interestingly only a few of the students indicated tutorials as a preferred tool for learning anatomy. About 7.65 of the students indicated they prefer to use computer-aided programs/learning (CAL) and interactive multimedia resources as indicated in Table 4.

## 4. Discussion

Anatomical knowledge is very crucial to the practice of medicine; thus its acquisition should not be left to the pedagogy of the day. The present study is to investigate student perceptions with regard to positive and negative aspects of cadaveric dissection in a PBL-based medical school in Ghana. Generally, the majority of the students held positive perceptions about the usefulness of cadaveric dissections in the teaching and learning of anatomy. The students held a strong perception that cadaveric dissection deepens their understanding of anatomy, provides a better understanding of clinical examination skills, enhances their respect towards the human body, and provides a better understanding of the effect of trauma as well as making learning interesting. There was also a general view that dissection helps students to recall what they have learned and gives them lasting knowledge. This perception expressed by the students in this study is similar to those expressed in a study by Dissabandara et al. [15] in Australia. Additionally, Izunya et al. [17] also stated that about 90% of the participants in their study considered cadaveric dissection as important and indispensable in the study of human anatomy. The results of the present study mean that notwithstanding the fact that the time for cadaveric dissection has been drastically reduced in an integrated PBL curriculum such us ours they still think dissection is very important in the teaching and learning of anatomy. Previous studies have shown that even though the utility of anatomy dissection in the modern medical curriculum is being questioned, there is evidence to show that those who took part in cadaveric dissection performed well in both written and oral exams [15, 18]. According to [19, 20] cadaveric dissections encourage deeper learning experience by providing a significant opportunity for students to study the exact nature of human tissues and their clinical relevance.

The major perceived disadvantages associated with cadaveric dissection in this study were the stressful nature of dissection and the smell of the formalin. In addition, the students indicated that it was difficult to locate structures. However, the majority of the students unanimously agreed that they would have been disadvantaged if they did not participate in the dissection programme. This finding is consistent with results of other studies. In several studies [14, 17, 21, 22] most students experienced anxiety and stress which impact negatively on their learning. According to Dissabandara et al. [15] some of the disadvantages perceived by students are the difficulty in finding and exploring structures during dissection as well as the stress associated with dissection. The students did not, however, feel that both their culture and religious inclination are against dissection.

In an integrated PBL curriculum such as ours, there is competition for time from other equally important components of the curriculum such as skills training, tutorials, and self-study. This significantly reduces the time allocated for dissection and students are expected to complete the session within a short period, thus making the process very stressful. The difficulty in identifying structures is characteristic with students who are being exposed to dissection for the first time. However, this problem is expected to facilitate critical thinking among students, which is an important component of the PBL system. Studies have shown that such problems associated with dissection are usually alleviated by adequate predissection preparation using lectures, model-based session, and proper tutor guidance during the dissection session.

Earlier studies on the methods of teaching and learning anatomy compared to dissection have recorded mixed results [20]. In the current study, however, the majority (53.0% of year 2 and 48.5% of year 3) of the students perceived dissection as a better way to learning anatomy over other forms of teaching and learning anatomy such as lectures, tutorials computer-assisted learning, and interactive multimedia learning applications. This is higher than the findings in a study by Dissabandara [15] where only 30% of their respondents said they prefer dissection to other forms of learning. The finding of the present study is, however, lower compared to the work of Izunya et al. [17] in which they found that about 84% of Nigerian medical students prefer dissection as the method of learning anatomy. There is a general perception among most Ghanaian medical students that one cannot be a very good medical doctor if you did not perform the cadaveric dissection. Among the six medical schools in Ghana currently, only the School of Medicine and Health Sciences in UDS uses the classical integrated PBL methodology in the training of medical students. The other medical schools use the traditional medical training methods where students have much time for dissection in the preclinical years. We speculate that this could also have influenced the perception of our students in choosing dissection as the preferred mode of acquiring anatomy knowledge.

## 5. Conclusion 

This study has shown a strong positive perception towards the use of cadaveric dissections in teaching and learning anatomy despite indicating that they did not like the smell of the formalin as well as the stressful nature of dissection regardless of the fact that SMHS/UDS uses the classical integrated PBL approach which gives limited time to dissections. Similarly, the students also think that dissection helps them to recall what they have learned and gives them lasting knowledge and they will, therefore, prefer dissection over other forms of teaching and learning anatomy.

## Figures and Tables

**Table 1:** Student perceptions about the importance of dissection.

Questionnaire Item	**Year 2**					**Year 3**				
	Frequency (%)					Frequency (%)				
	SD	D	N	A	SA	SD	D	N	A	SA
Overall, I am satisfied with the dissection program	2(1.5)	8(6.0)	20(15.2)	68(51.5)	34(25.8)	6(4.5)	34(25.8)	28(21.2)	36(27.3)	28(21.2)
The dissection deepened my understanding of anatomy	0(0.0)	4(3.0)	14(10.6)	86(65.2)	30(22.74(3.0)	4(3.0)	6(4.5)	24(18.2)	68(51.5)	30(22.7)
The dissection provides better understanding of clinical skills examination	2(1.5)	10(7.6)	32(24.2)	62(47.0)	26(19.7)	2(1.5)	16(12.1)	22(16.7)	60(45.5)	32(24.2)
The dissection enhanced my respect towards the human body	4(3.0)	12(9.1)	28(21.2)	56(42.4)	32(24.2)	2(1.5)	4(3.0)	22(16.7)	68(51.5)	36(27.3)
The dissection provided better understanding of the effect of trauma	4(3.0)	2(1.5)	36(27.3)	58(43.9)	34(25.8)	0(0.0)	10(7.8)	36(27.3)	54(40.9)	30(22.7)
Dissection makes learning more interesting	2(1.5)	0(0.0)	12(9.1)	76(57.6)	44(33.3)	0(0.0)	2(1.5)	12(9.1)	68(51.5)	50(37.9)
The dissection helped me to recall what I learnt	0(0.0)	4(3.0)	12(9.1)	80(60.6)	36(27.3)	0(0.0)	6(4.5)	28(21.2)	60(45.5)	36(27.3)
Dissections gives me a lasting knowledge	0(0.0)	8(6.1)	20(15.1)	86(65.2)	18(13.6)	0(0.0)	10(7.6)	32(24.2)	62(47.0)	28(21.2)

**Table 2:** Students perception about the disadvantages of dissection.

Questionnaire Item	**Year 2**					**Year 3**				
	Frequency (%)					Frequency (%)				
	SD	D	N	A	SA	SD	D	N	A	SA
It was difficult locating structures	4(3.0)	0(0.0)	32(24.2)	78(59.1)	18(13.6)	6(4.6)	0(0.0)	30(22.7)	58(43.9)	38(28.8))
I could not differentiate between structures	6(4.5)	48(36.4)	36(27.3)	30(22.7)	12(9.1)	0(0.0)	38(28.8)	36(27.3)	38(28.8)	20(15.2)
I was bored with the way it was carried out	24(18.2)	56(42.4)	36(27.3)	12(9.1)	4(3.0)	14(10.6)	32(24.4)	30(22.7)	30(22.7)	26(19.7)
The dissection was very stressful	4(3.0)	40(30.3)	28(21.2)	46(34.9)	14(10.6)	10(7.6)	22(16.7)	24(18.2)	44(33.3)	32(24.2)
The dissection was time consuming	12(9.1)	70(53.0)	24(18.2)	24(18.2)	2(1.5)	20(15.2)	50(37.9)	22(16.7)	22(16.7)	16(12.1)
I did not like the smell of formalin	8(6.1)	12(9.1)	30(22.7)	48(36.4)	30(22.7)	10(7.6)	18(13.6)	28(21.2)	40(30.3)	36(27.3)
I feel dissection is against my culture	86(65.2)	42(31.8)	4(43.0)	0(0.0)	0(0.0)	72(54.5)	40(30.3)	18(13.6)	0(0.0)	2(1.5)
I feel dissection is against my religion	80(60.6)	46(34.8)	6(4.5)	0(0.0)	0(0.0)	76(57.6)	30(22.7)	24(18.2)	0(0.0)	2(1.5)
I think dissection should be eliminated from the curriculum	100(75.8)	30(22.7)	2(1.5)	0(0.0)	0(0.0)	112(84.8)	20(15.2)	0(0.0)	0(0.0)	0(0.0)

**Table 3:** Comparison of dissection to other forms of learning.

Questionnaire Item	Year 2					Year 3				
	Frequency (%)					Frequency (%)				
	SD	D	N	A	SA	SD	D	N	A	SA
Dissection should be replaced by Lectures	100(75.8)	30(22.7)	2(1.5)	0(0.0)	0(0.0)	98(74.2)	26(19.7)	4(3.0)	0(0.0)	4(3.0)
I feel Dissection should be replaced by computer-based programs	78(59.1)	36(27.3)	16(12.1)	2(1.5)	0(0.0)	68(51.5)	50(37.9)	10(7.6)	(1.5)	4(3.0)
Dissection should be replaced by pre-dissected material	54(40.9)	42(31.8)	32(24.2)	4(3.0)	0(0.0)	56(42.4)	44(33.3)	20(15.2)	4(3.0)	4(3.0)
I prefer dissection classes over other forms of learning	0(0.0)	20(15.2)	32(24.2)	60(45.5)	20(15.2)	4(3.0)	16(12.1)	38(28.8)	60(45.5)	14(10.6)
More time should be allocated to dissection	0(0.0)	20(15.2)	36(27.3)	48(36.4)	28(21.2)	2(1.5)	16(12.1)	20(15.2)	60(45.5)	34(25.8)
Students should be allowed to perform dissections by themselves	4(3.0)	10(7.6)	30(22.7)	40(30.3)	48(36.4)	6(4.6)	12(9.1)	20(15.2)	42(31.8)	52(39.4)
I will be disadvantaged if I do not attend dissection	2(1.5)	6(4.6)	2(1.5)	66(50.0)	56(42.4)	0(0.0)	2(1.5)	16(12.1)	54(40.9)	60(45.5)

**Table 4:** Preferred tools for learning anatomy by the students.

**Tools for Learning Anatomy**	**Year 2** **N (**%**)**	**Year 3** **N (**%**)**
Lectures	20 (15.1)	28 (21.2)
Self-study	14 (10.6)	18 (13.6)
Dissection	70 (53.0)	64 (48.5)
Tutorials	6 (4.6)	4 (3.0)
CAL programs	10 (7.6)	6 (4.6)
Interactive Multimedia Resources	10 (7.6)	10 (7.6)
Peer Learning	2 (1.5)	2 (1.5)
**Total**	132 (100.0)	132 (100.0)

## Data Availability

The data used to support the findings of this study are available from the corresponding author upon request.
